# The powdery mildew-resistant Arabidopsis *mlo2 mlo6 mlo12* triple mutant displays altered infection phenotypes with diverse types of phytopathogens

**DOI:** 10.1038/s41598-017-07188-7

**Published:** 2017-08-24

**Authors:** Johanna Acevedo-Garcia, Katrin Gruner, Anja Reinstädler, Ariane Kemen, Eric Kemen, Lingxue Cao, Frank L. W. Takken, Marco U. Reitz, Patrick Schäfer, Richard J. O’Connell, Stefan Kusch, Hannah Kuhn, Ralph Panstruga

**Affiliations:** 1 RWTH Aachen University, Institute for Biology I, Unit of Plant Molecular Cell Biology, Worringerweg 1, 52074 Aachen, Germany; 2 Max Planck Institute for Plant Breeding Research, Carl-von-Linné-Weg 10, 50829 Cologne, Germany; 3 University of Amsterdam, Swammerdam Institute for Life Sciences, Molecular Plant Pathology, Science Park 904, 1098 XH Amsterdam, The Netherlands; 4 University of Warwick, The School of Life Sciences, Gibbet Hill Campus, Coventry, CV4 7AL UK; 5 UMR BIOGER, INRA, AgroParisTech, Université Paris-Saclay, 78850 Thiverval-Grignon, France

## Abstract

*Arabidopsis thaliana mlo2 mlo6 mlo12* triple mutant plants exhibit complete immunity against infection by otherwise virulent obligate biotrophic powdery mildew fungi such as *Golovinomyces orontii*. While this phenotype is well documented, the interaction profile of the triple mutant with other microbes is underexplored and incomplete. Here, we thoroughly assessed and quantified the infection phenotypes of two independent powdery mildew-resistant triple mutant lines with a range of microbes. These microorganisms belong to three kingdoms of life, engage in diverse trophic lifestyles, and deploy different infection strategies. We found that interactions with microbes that do not directly enter leaf epidermal cells were seemingly unaltered or showed even enhanced microbial growth or symptom formation in the *mlo2 mlo6 mlo12* triple mutants, as shown for *Pseudomonas syringae* and *Fusarium oxysporum*. By contrast, the *mlo2 mlo6 mlo12* triple mutants exhibited reduced host cell entry rates by *Colletotrichum higginsianum*, a fungal pathogen showing direct penetration of leaf epidermal cells comparable to *G. orontii*. Together with previous findings, the results of this study strengthen the notion that mutations in genes *MLO2*, *MLO6* and *MLO12* not only restrict powdery mildew colonization, but also affect interactions with a number of other phytopathogens.

## Introduction

Powdery mildew is a widespread fungal disease of many angiosperm plants^1^. It is caused by ascomycetes of the order Erysiphales. The more than 400 known powdery mildew species can infect ~10,000 plant species, including the dicotyledonous reference species *Arabidopsis thaliana* ^2^. Currently, four powdery mildew species have been reported to be able to complete their asexual life cycle on Arabidopsis host plants, i.e. *Erysiphe cruciferarum*, *Golovinomyces cichoracearum*, *G. orontii* and *Oidium neolycopersici* ^3, 4^.

The major naturally occurring source of resistance effective against powdery mildews in Arabidopsis is *RESTANCE TO POWDERY MILDEW8* (*RPW8*)^5–7^. This complex locus shows extensive intraspecific genetic variation and confers dominantly inherited resistance against multiple powdery mildew species. The respective genes encode non-canonical resistance proteins that lead to arrest of fungal pathogenesis after host cell penetration (“post-penetration resistance”). Effective resistance correlates with the encasement of the fungal feeding structures (haustorial complexes) in a callose-containing cell wall matrix^8^.

A different type of powdery mildew resistance is conferred by recessively inherited loss-of-function mutations in specific *MILDEW RESISTANCE LOCUS O* (*MLO*) genes. These genes, which encode integral membrane proteins of unknown biochemical activity, comprise a family of 15 members in Arabidopsis^9^. Loss-of-function mutations in *MLO2* (At1g11310) result in incomplete resistance against powdery mildew attack that is characterized by a reduction in host cell entry rates by ~50%. This coincides with an arrest of hyphal growth prior to the formation of conidiophores in *mlo2* plants, resulting in almost entirely abolished sporulation^10, 11^. Mutations in *MLO6* (At1g61560) and *MLO12* (At2g39200) do not affect powdery mildew interactions on their own. However, they co-operatively enhance *mlo2*-conditioned resistance, and in combination with a mutation in *MLO2* cause a complete lack of host cell penetration by fungal sporelings, leading to complete immunity (“pre-penetration resistance”). This type of powdery mildew resistance is best known from barley, where natural and induced *mlo* mutants have been discovered more than 70 years ago and have been successfully employed in agriculture for over 35 years^12, 13^. More recently, *mlo*-based resistance was described in several other monocotyledonous and dicotyledonous plant species such as, amongst others, pea^14^, tomato^15^ and wheat^16, 17^. Hence, *mlo*-based resistance is a seemingly universal phenomenon within angiosperm plant species that are hosts to powdery mildew fungi^18^.

At the phenotypical and molecular level, *mlo* resistance resembles the highly effective defence against non-adapted powdery mildews (“non-host resistance”^19^). The *mlo2 mlo6 mlo12* mutant shows a spectacular level of resistance against different powdery mildews^11, 15^. Conversely, it revealed slightly enhanced disease symptoms–and in part also pathogen proliferation–in interactions with some hemibiotrophic/necrotrophic pathogens such as *Alternaria alternata*, *A. brassicicola* and *Phytophthora infestans* ^11^. These phenotypes might be the indirect consequence of deregulated mesophyll cell death in leaves of the *mlo2 mlo6 mlo12* mutant. Similar to the barley *mlo* mutant, leaves of the triple mutant are subject to spontaneous deposition of callose-containing cell wall appositions and ultimately premature senescence^11, 20–22^.

Apart from powdery mildews, the model plant Arabidopsis can serve as a host for a number of additional microbial pathogens and/or endophytes. Well-established and widely studied patho-systems comprise the interaction of Arabidopsis with bacteria (e.g. *Pseudomonas syringae* ^23^) and oomycetes (e.g. *Hyaloperonospora arabidopsidis* ^24^ and *Albugo* spp.^25, 26^), which cause the bacterial speck, downy mildew and white rust disease, respectively. More recently established patho-systems include, amongst others, the interaction of Arabidopsis with the anthracnose fungus *Colletotrichum higginsianum* ^27^ and the causal agent of the vascular wilt and root rot disease, *Fusarium oxysporum* ^28, 29^. Apart from pathogenic microbes, Arabidopsis harbors a broad range of bacterial and fungal endophytes in the rhizo- and phyllosphere when grown under natural conditions (leaf and root microbiota and mycobiota)^30, 31^. A well-studied root-colonizing endophyte of many plant species, including Arabidopsis, with a reported growth-promoting activity is *Serendipita indica* (syn: *Piriformospora indica*)^32–34^.

While the role of *mlo* mutants in providing resistance to powdery mildew fungi is well-documented^18^, the effect of mutations in *MLO* genes on colonization with other microbes has not yet been explored systematically in a quantitative manner. To fill this existing gap in knowledge, we performed comprehensive infection assays with two independent powdery mildew-resistant *mlo2 mlo6 mlo12* T-DNA knockout lines of Arabidopsis and a range of microbial species that exhibit different lifestyles and diverse modes of plant colonization. Quantitative assessment in relation to control genotypes revealed altered infection phenotypes of the *mlo2 mlo6 mlo12* mutants in the case of the fungal parasites *F. oxysporum* and *C. higginsianum* and the bacterial pathogen *P. syringae*.

## Results

In order to establish a comprehensive interaction profile of the Arabidopsis *mlo2 mlo6 mlo12* triple mutant, we challenged individuals of two independent triple mutant lines with a broad panel of microorganisms that are known to be virulent on the Arabidopsis Col-0 accession. These two mutant lines represent two entirely independent allele combinations in the genetic background of Col-0. The resulting infection phenotypes were compared with that of Col-0 wild type, which served as control in all experiments. At the taxonomic level, the panel of tested microorganisms comprised fungal (*G. orontii*, *F. oxysporum*, *C. higginsianum*, *S. indica*), oomycete (*H. arabidopsidis*, *A. laibachii*) and bacterial (*P. syringae*) species, which engage in obligate biotrophic (*G. orontii*, *H. arabidopsidis*, *A. laibachii*), and hemibiotrophic (*P. syringae*, *C. higginsianum* and *F. oxysporum*) interactions, respectively. We also included in our set of experiments a fungal endophyte (*S. indica*) that is known to have a growth-promoting effect on colonized Arabidopsis plants.

### A novel *mlo2 mlo6 mlo12* triple mutant line with near-complete powdery mildew resistance

**Figure 1:**
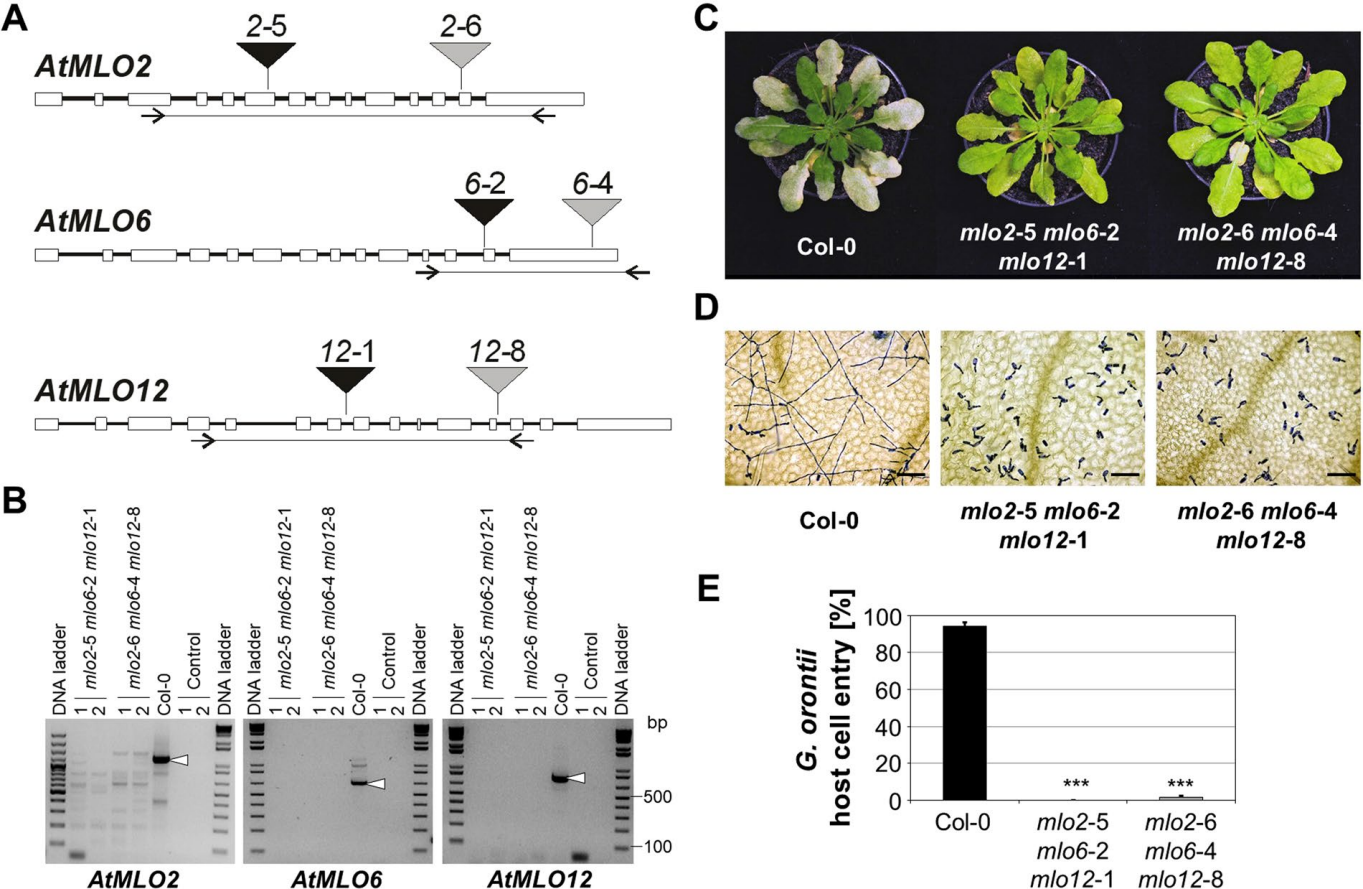
The *G. orontii* resistance phenotype of the *mlo2*-6 *mlo6*-4 *mlo12*-8 triple mutant is indistinguishable from the *mlo2*-5 *mlo6*-2 *mlo12*-1 triple mutant. Six-week-old Arabidopsis plants were touch-inoculated with *G. orontii* conidiospores. (**A**) Scheme depicting the T-DNA insertion sites in *MLO2*, *MLO6* and *MLO12*. Rectangles represent exons, black lines introns. Triangles symbolize the T-DNA insertion sites of the various *mlo* alleles. Lines flanked by inverted arrows (primer binding sites) below the gene models indicate the RT-PCR amplicons used to test for *MLO* transcript accumulation in the mutant lines. (**B**) RT-PCR analysis of *MLO2*, *MLO6* and *MLO12* transcript accumulation. Primer pairs covering the regions indicated in panel A were used to amplify the respective transcript amplicons from cDNA of lines *mlo2*-5 *mlo6*-2 *mlo12*-1 and *mlo2*-6 *mlo6*-4 *mlo12*-8 (two individuals each) as well as Col-0 wild-type plants (positive control). RT-PCR reactions without reverse transcription (control 1) and amplification without template (control 2) served as negative controls. White arrowheads indicate RT-PCR products of the expected size in case of Col-0 wild-type plants. (**C**) Representative macroscopic infection phenotypes at 8 dpi. (**D)** Light micrographs visualizing fungal pathogenesis at 48 hpi. Leaf samples were cleared in destaining solution and fungal infection structures subsequently stained with Coomassie Brillant Blue. Bars = 100 µm. (**E**) Quantitative assessment of host cell entry. Data show the mean ± standard error of the mean (SEM) from three experiments. In each experiment, at least 100 interaction sites from 1-3 leaves of five independent plants per genotype were assessed (total of > 500 interaction sites per genotype and experiment). *** Indicates a statistically significant difference from Col-0 (*P* < 0.001) according to a GLM test (binomial distribution) on n = 3 independent experimental replicates.

We previously showed that the triple mutant line *mlo2*-5 *mlo6*-2 *mlo12*-1 is fully resistant to the adapted powdery mildew pathogen, *G. orontii* ^11^. We generated a second triple mutant line, *mlo2*-6 *mlo6*-4 *mlo12*-8, which is based on different T-DNA insertions in the three *AtMLO* genes (Fig. 1A; see Materials and Methods for further details). As revealed by reverse transcriptase-polymerase chain reaction (RT-PCR) analysis, the T-DNA insertions in these lines result in a lack of full-length *MLO2*, *MLO6* and *MLO12* transcripts (Fig. 1B). The two triple mutant lines grow similarly as Col-0 wild-type plants, but suffer from early leaf senescence as described before in detail for *mlo2*-5 *mlo6*-2 *mo12*-1^11, 20^. This phenotype is evident by the slightly chlorotic rosette leaves of the two *mlo2 mlo6 mlo12* lines at the age of approximately six weeks (Fig. 1C) We analysed the triple mutants upon challenge with *G. orontii* and found that line *mlo2*-6 *mlo6*-4 *mlo12*-8 fully resembles line *mlo2*-5 *mlo6*-2 *mlo12*-1 with respect to the macroscopic and microscopic infection phenotypes (Fig. 1C–E). Unlike the Col-0 control plants, which showed abundant fungal sporulation at 8 days post inoculation (dpi), individuals of both lines lacked visible powdery mildew symptoms (Fig. 1C). This finding was consistent with analysis at the microscopic level at 48 hours post inoculation (hpi), which revealed an early abortion of fungal pathogenesis at the level of host cell entry in the two triple mutants, while Col-0 control plants showed extensive mycelial growth (Fig. 1D). We noted, however, that line *mlo2*-5 *mlo6*-2 *mlo12*-1 was entirely resistant, lacking any recognizable host cell penetration (0% entry rate as judged by the absence of secondary hyphae and discernible haustoria) in our experiments, while line *mlo2*-6 *mlo6*-4 *mlo12*-8 allowed the occasional formation of fungal micro-colonies (ca. 1% entry rate; Fig. 1E). With our sample size, this minor difference between the two *mlo2 mlo6 mlo12* genotypes was not statistically significant. Thus, the two *mlo2 mlo6 mlo12* triple mutants are essentially equivalent with regard to the level of resistance against the obligate biotrophic powdery mildew pathogen, *G. orontii*.

### *mlo2 mlo6 mlo12* plants show unaltered susceptibility to downy mildew

**Figure 2:**
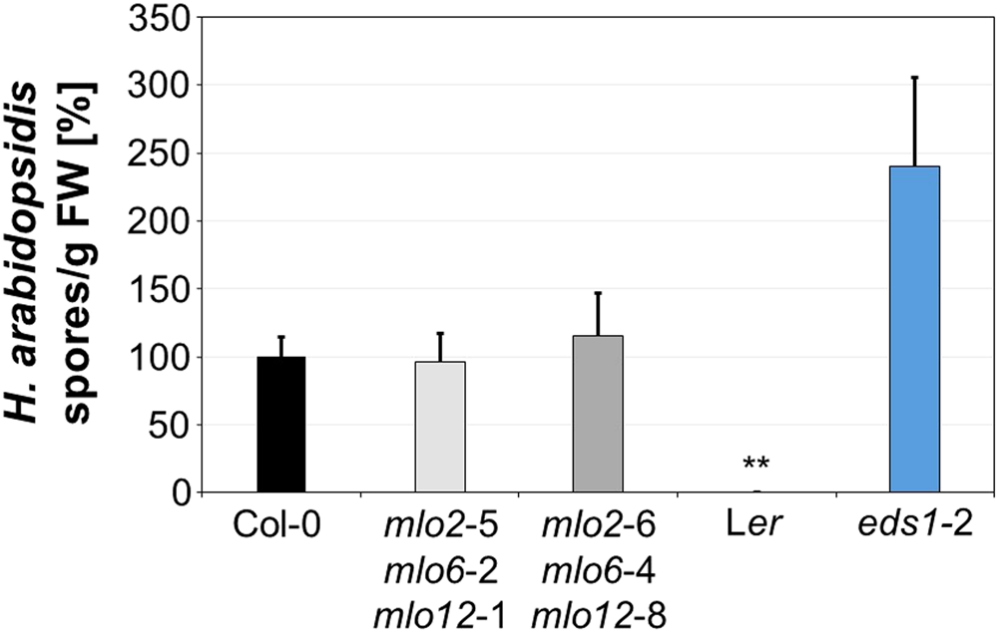
The *mlo2 mlo6 mlo12* triple mutants show an unaltered *H. arabidopsidis* phenotype. Sixteen-day-old seedlings were spray-inoculated with *H. arabidopsidis* (isolate Noco2) and spore formation (spores per g FW) was quantitatively assessed at 7 dpi. Sporulation was normalized relative to accession Col-0 set as 100%. Data show the mean ± SEM based on five independent experiments. Accession L*er *served as resistant control, the mutant *eds1*-2 as super-susceptible control. ** Indicates a statistically significant difference from Col-0 (*P* < 0.01) according to a Wilcoxon-Mann-Whitney rank sum test on n = 5 independent experimental replicates.

We next assessed the infection phenotype of the *mlo2 mlo6 mlo12* triple mutants with the obligate biotrophic oomycete *H. arabidopsidis* (isolate Noco2) upon spray inoculation of 16-day-old seedlings. At 7 dpi, we scored the formation of conidiospores in comparison to the susceptible accession Col-0, the resistant accession Landsberg *erecta* (L*er*) and the super-susceptible *eds1*-2 mutant^35, 36^ (in Col-0 genetic background) using a hemocytometer-based assay. Owing to considerable experiment-to-experiment variation regarding the absolute numbers of conidiospores per gram seedlings, we normalized the results of the five independent experiments to the values obtained with Col-0, set as 100%. In comparison to Col-0 (100% ± 15%), the two *mlo2 mlo6 mlo12* triple mutants showed unaltered levels of conidiospore formation (96% ± 21% and 115% ± 32%, respectively; no statistically significant difference from Col-0), while L*er*(0% ± 0%) and the *eds1*-2 mutant (240% ± 66%) revealed the expected resistant and super-susceptible phenotypes (Fig. 2). These data demonstrate that the infection phenotype with *H. arabidopsidis* isolate Noco2 is unaltered in the *mlo2 mlo6 mlo12* triple mutants.

### *mlo2 mlo6 mlo12* plants show unaffected susceptibility to white rust

**Figure 3:**
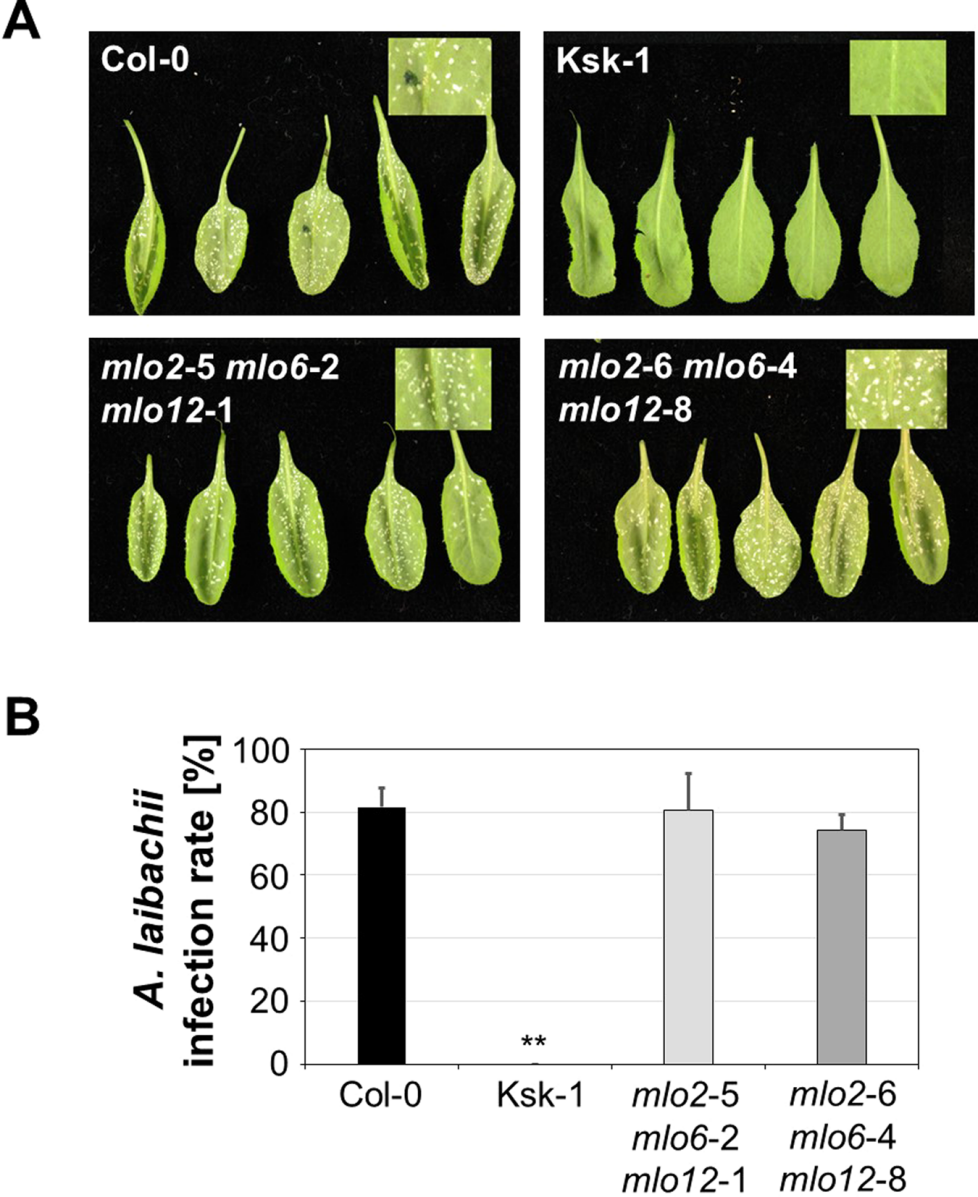
The *mlo2 mlo6 mlo12* triple mutants show an unaltered *A. laibachii* infection phenotype. Six-week-old plants were spray-inoculated with *A. laibachii* (isolate Nc14) and infection phenotypes were assessed at 10 dpi. (**A**) Representative examples of inoculated rosette leaves of accessions Col-0, Ksk-1 and the two *mlo2 mlo6 mlo12* triple mutant lines. Insets show a magnification of an inoculated leaf area. (**B**) Quantitative evaluation of the infection rate. Data show the mean ± standard deviation (SD) of the proportion of rosette leaves with disease symptoms (macroscopically visible white rust pustule formation) based on 5-6 plants per genotype and 12-30 evaluated leaves per plant. ** Indicates a statistically significant difference from Col-0 (*P* < 0.01) according to a Wilcoxon-Mann-Whitney rank sum test.

We then analysed the interaction of the *mlo2 mlo6 mlo12* triple mutants with another obligate biotrophic oomycete, *A. laibachii* (white rust pathogen; isolate Nc14), following spray inoculation with zoospores. The susceptible accession Col-0 and the resistant accession Keswick (Ksk-1^37^) were included as controls. At 10 dpi, infection phenotypes were scored (Fig. 3A) and infection rates were quantitatively assessed as the percentage of uninfected (showing no signs of white rust sporulation) *versus* infected (showing abundant sporulation) leaves. This experiment revealed infection rates of 82% ± 6% for Col-0 and 0% ± 0% for Ksk-1, compared to 81% ± 12% for *mlo2*-5 *mlo6*-2 *mlo12*-1 and 74% ± 5% for *mlo2*-6 *mlo6*-4 *mlo12*-8 (Fig. 3B). The values for the two *mlo2 mlo6 mlo12* triple mutants were not significantly different from the Col-0 control plants, as supported by our statistical analysis. Taken together, the data indicate that the susceptibility to *A. laibachii* does not differ between Col-0 and the two *mlo2 mlo6 mlo12* triple mutants.

### *mlo2 mlo6 mlo12* plants show enhanced resistance to *C. higginsianum*

**Figure 4:**
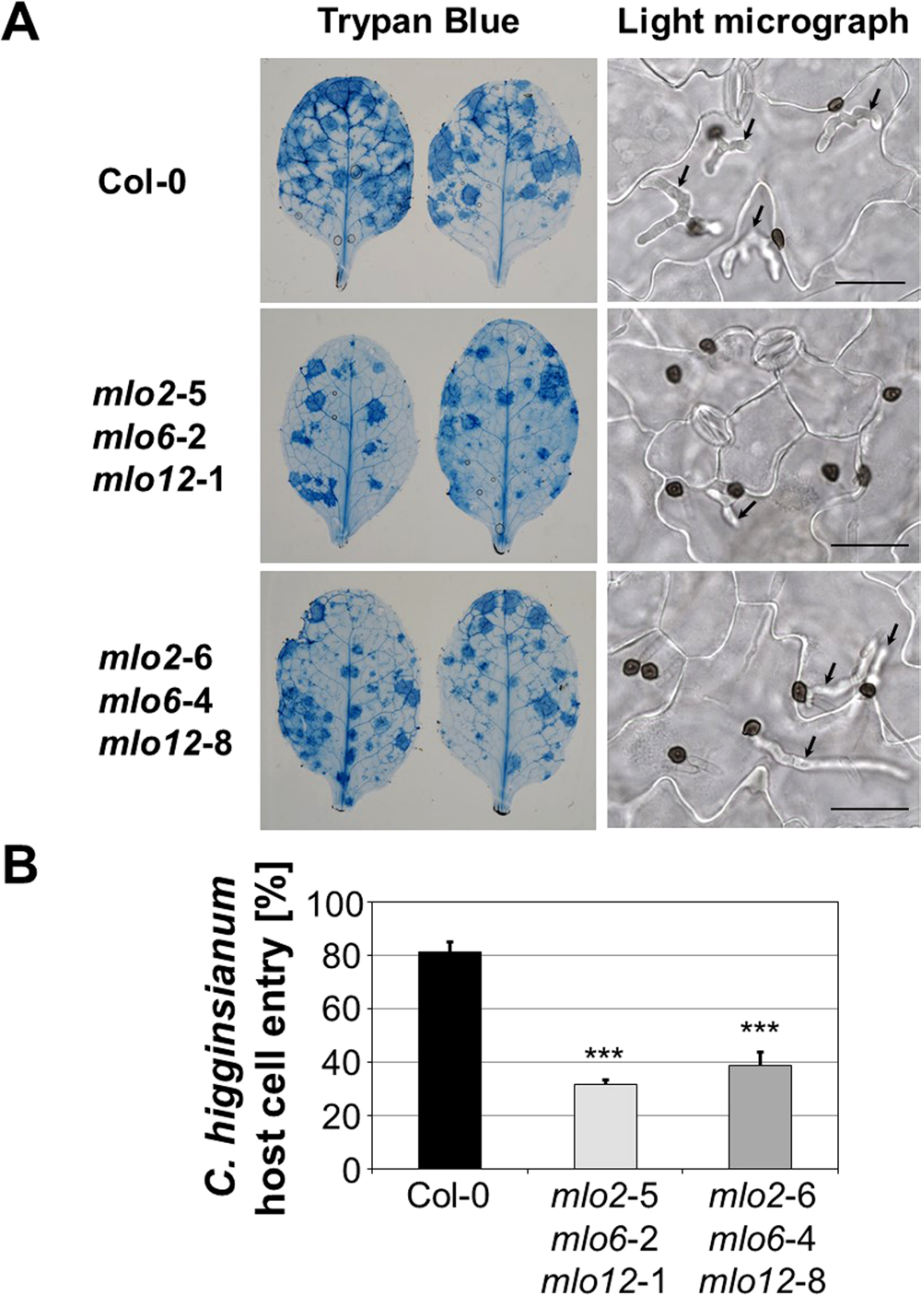
The *mlo2 mlo6 mlo12* triple mutants show decreased host cell entry by *C. higginsianum*. Infection phenotypes of Col-0, *mlo2-*5 *mlo6-*2 *mlo12-*1 and *mlo2-*6 *mlo6-*4 *mlo12-*8 at 3 dpi with *C. higginsianum* (isolate IMI349063A). Plants were spray-inoculated with spore suspension (5 × 10^5^ spores ml^−1^). (**A**) Representative examples of whole leaves cleared and stained with Trypan blue (left column) and light micrographs showing leaf epidermal cells after clearing in choral hydrate (right column). Biotrophic hyphae (arrows) are visible beneath some melanised appressoria. Bars = 30 μm. (**B**) Quantitative assessment of host cell entry. Data show the mean ± SD from counts of at least 140 appressoria from each leaf (one leaf from each of 3 different plants), i.e. at least 420 appressoria per plant genotype. ***Indicates a statistically significant difference from Col-0 (*P* < 0.001) according to a GLM test (Poisson distribution) on n = 3 technical replicates (individual plants). The experiment was repeated once with similar results (Fig. S1).

We further extended our study by analysing infection of the *mlo2 mlo6 mlo12* triple mutants with the hemibiotrophic ascomycete pathogen *C. higginsianum* (strain IMI349063). At 3 dpi, the foliar fungal pathogen triggered local host cell death (necrotic lesions) in rosette leaves of *mlo2 mlo6 mlo12* plants, the extent of which was seemingly lower in the mutants than in Col-0 control plants (Fig. 4A). Consistent with this observation, quantitative cytological analysis at 3 dpi revealed reduced levels of host cell entry (successful host cell penetration and formation of intracellular biotrophic hyphae) in both *mlo2 mlo6 mlo12* triple mutant lines compared to Col-0 control plants (Fig. 4B). While both mutant lines showed a comparable decrease in host cell entry in this experiment, there was a statistically significant difference between the two triple mutant lines in a second independent experiment (Fig. S1). Taken together, both host cell penetration and symptom formation are reduced in both *mlo2 mlo6 mlo12* mutant lines.

### *mlo2 mlo6 mlo12* plants show enhanced disease symptoms to *F. oxysporum*

**Figure 5:**
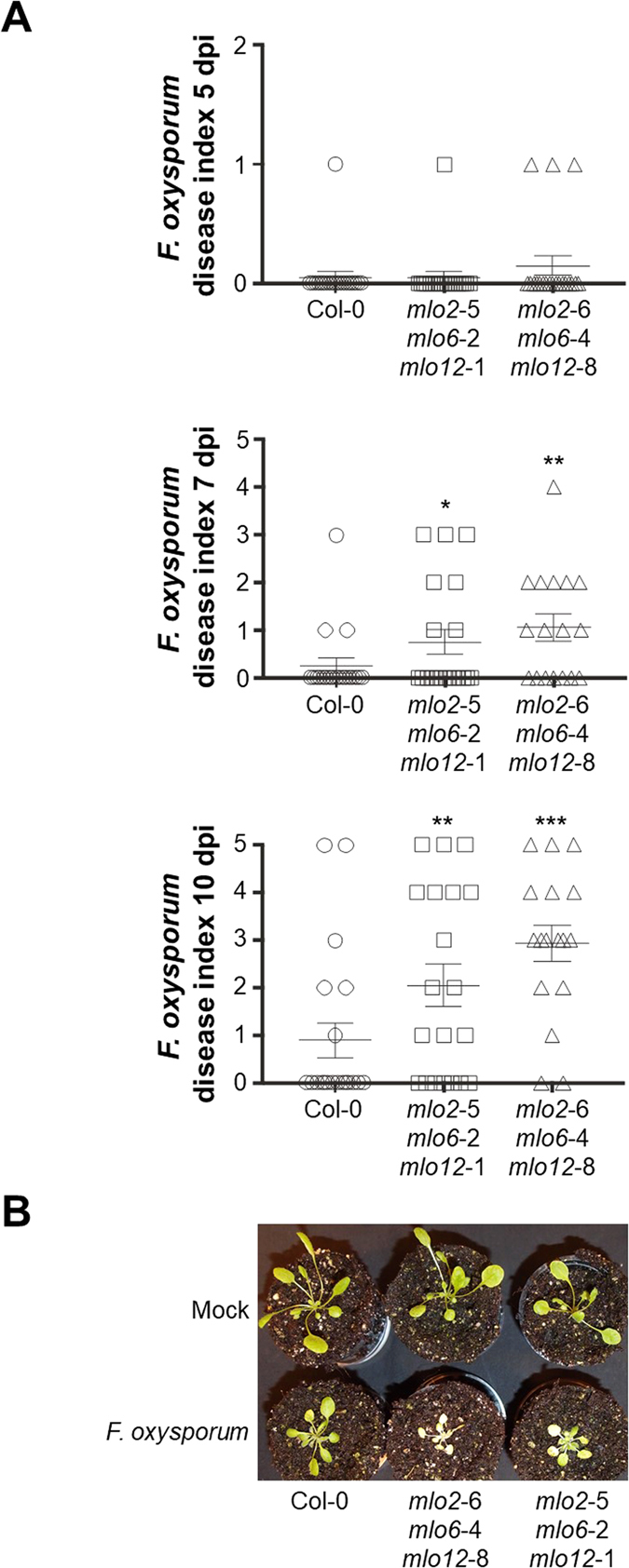
The *mlo2 mlo6 mlo12* triple mutants show enhanced disease symptoms upon challenge with *F. oxysporum*. Two-week-old Arabidopsis seedlings were inoculated with a spore suspension of *F. oxysporum* (isolate Fo5176). (**A**) Infection phenotypes were scored at 5, 7 and 10 dpi by assigning a disease index on a 0 (no symptoms) to 5 (severe disease symptoms) scale. Data shown are from a representative experiment and based on 15-20 seedlings per genotype. Each symbol in the categorical scatter plot (circle, square or triangle) represents the infection phenotype of one seedling. The crosses indicate the mean values ± SEM. *, ** and *** indicate statistically significant differences from Col-0 (*P* < 0.05, *P* < 0.01 and *P* < 0.001, respectively) according to a GLM test (Poisson distribution) on n = 15-20 technical replicates (individual seedlings). The experiment was repeated twice with similar results (Fig. S2). (**B**) Representative macroscopic infection phenotypes at 7 dpi.

We then turned our attention to the hemibiotrophic root-infecting fungal pathogen *F. oxysporum*. Following dip inoculation of roots of 2-week-old plants in spore suspensions of *F. oxysporum* isolate Fo5176, disease progression was scored at 5, 7 and 10 dpi by semi-quantitative classification of disease symptoms into six categories and by calculating an “average disease index” based on this classification^38^. Fifteen to forty plants were assessed per experimental replicate, genotype and time point. This experimental setup revealed more severe disease symptoms in the two *mlo2 mlo6 mlo12* mutants, typically recognizable as a tendency already at 5 dpi, becoming more evident at 7 dpi and being pronounced at 10 dpi (Figs 5 and S2). Taking into account all three experimental replicates, there was no apparent difference in hypersusceptibility between the two *mlo2 mlo6 mlo12* mutant lines.

### *mlo2 mlo6 mlo12* plants show enhanced susceptibility to *P. syringae*

**Figure 6:**
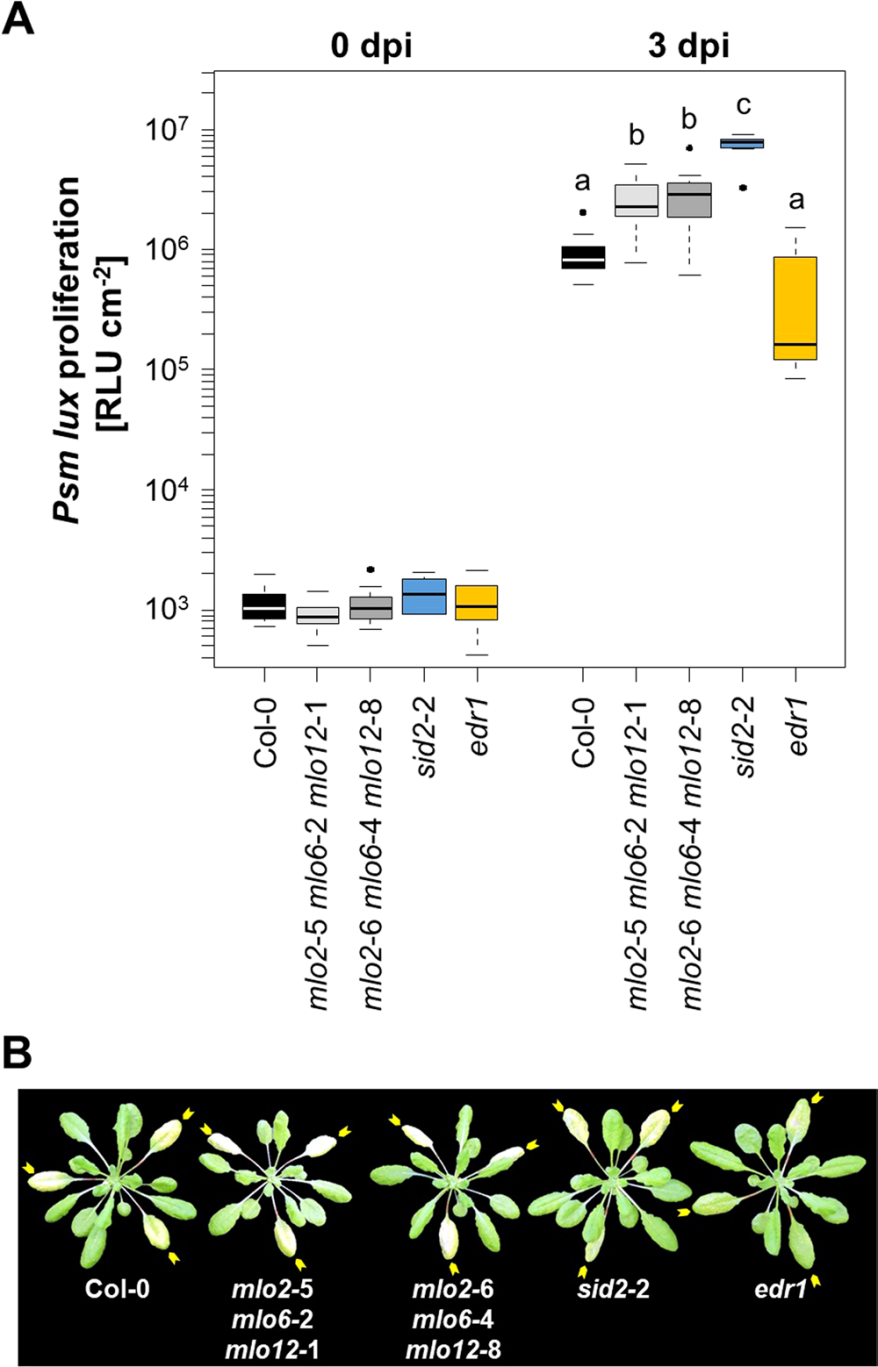
The *mlo2 mlo6 mlo12* triple mutants show an elevated bacterial titre upon challenge with *P. syringae*. Five-week-old Arabidopsis plants were infiltrated with *P. syringae* pv. *maculicola lux* (OD_600_ = 0.0005) and luminescence (RLU cm^−2^; corresponding to bacterial titre) determined at 0 dpi (to ensure an equal start inoculum) and 3 dpi. (**A**) The boxplot shows data from one representative experiment based on n = 7 (0 dpi) and n = 11 plants (3 dpi) per genotype, with each plant value representing the mean of three leaves. Centre lines mark the medians, upper and lower box limits indicate the 25^th^ and 75^th^ percentiles, respectively; upper and lower whiskers extend 1.5 times the interquartile range from the 25^th^ and 75^th^ percentiles, respectively; and dots represent outliners. Letters indicate statistically different groups (at least *P* < 0.05) according to a GLM test (quasi-Poisson distribution) on n = 11 technical replicates (individual plants). The experiment was repeated four times with n = 7-13 plants per genotype in each experiment and in two different controlled environments (see Materials and Methods for details) with comparable results (Fig. S3). (**B**) Representative macroscopic infection phenotypes at 3 dpi. Yellow arrows indicate the inoculated leaves.

Next, we examined the infection phenotype of the two *mlo2 mlo6 mlo12* triple mutant lines with the bacterial pathogen *P*. *syringae* pv. *maculicola* (strain ES4326). For these experiments, we deployed a strain that carries a chromosomal integration of the *Photorhabdus luminescens* luciferase gene (here designated as *P. syringae* pv. *maculicola* *lux*). Luminescence emitted by this strain reliably reports bacterial growth in infected Arabidopsis leaves^39^. We infiltrated bacterial suspensions into leaves of Col-0 wild type and *mlo2 mlo6 mlo12* mutant plants and further included *sid2*-2/*eds16*-1^40^ and *edr1* ^41^ mutants as additional control plants, which were previously reported to show super-susceptibility and enhanced resistance, respectively, in response to infection with *P. syringae*. Luminescence was recorded shortly after inoculation (0 dpi) and at 3 dpi. In the case of the two triple mutants, we observed increased luminescence at 3 dpi, corresponding to approximately two-fold higher bacterial titres compared to Col-0, while the *sid2*-2 and *edr1* controls showed the expected phenotypes of intensely increased susceptibility in *sid2-2* (approx. 8-fold increase) and a tendency to an increased resistance in *edr1* (Fig. 6). A slight but statistically significant increase in bacterial titres in the *mlo2 mlo6 mlo12* mutant plants was found in three out of four additional experimental replicates of this pathogen assay (Fig. S3). Taken together, both *mlo2 mlo6 mlo12* mutant lines show somewhat increased susceptibility to *P. syringae* pv. *maculicola*.

### *mlo2 mlo6 mlo12* plants show an unaltered infection phenotype with *S. indica*

**Figure 7:**
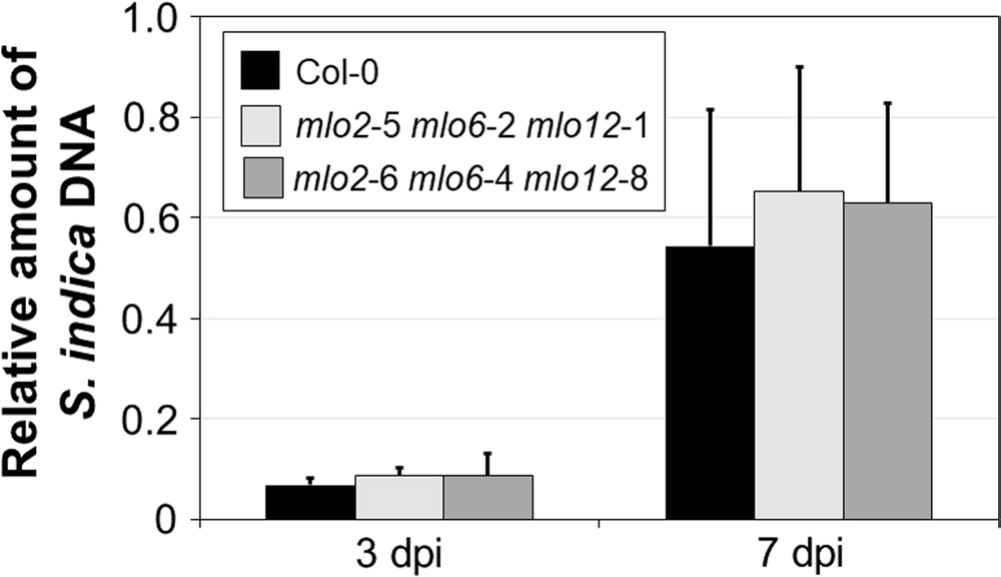
The *mlo2 mlo6 mlo12* triple mutants show unaltered colonization by *S. indica*. Roots of 2-week-old plants were inoculated with *S. indica* (isolate DSM11827) and the rate of root colonization determined by quantifying the relative amount of fungal genomic DNA by qPCR analysis. Data show the mean ± SEM based on three experiments with 240 seedlings per genotype and experiment. There is no statistically significant difference between genotypes according to GLM (quasi-Poisson distribution) on n = 3 independent experimental replicates.

We finally focused our attention on the fungal root endophyte *S. indica*, belonging to the Basidiomycota, which exerts a beneficial effect on the growth of Arabidopsis^32^. Interaction assays with this species (isolate DSM11827) were performed with sterile seedlings in petri dishes, and the extent of *S. indica* colonization was assessed relative to accession Col-0 *via* genomic DNA content by quantitative PCR analysis at 3 and 7 dpi^34^. Based on three independent experiments, we observed no statistically significant difference in fungal DNA content at any time point between Col-0 control plants and the two *mlo2 mlo6 mlo12* triple mutant lines (Fig. 7).

### *A. laibachii* cannot break powdery mildew resistance of *mlo2 mlo6 mlo12* plants

Although Arabidopsis is a nonhost plant for the oomycete *P. infestans*, the causal agent of late blight in tomato and potato, a recent study reports that pre-infection with *A. laibachii* can render Arabidopsis susceptible to colonization by *P. infestans* ^42^. Based on this finding, we wondered whether pre-infection with *A. laibachii* could also overcome *mlo*-based resistance in Arabidopsis and turn an otherwise incompatible interaction between *mlo2 mlo6 mlo12* triple mutants and the powdery mildew pathogen *G. orontii* into a compatible one. To test this speculation, we spray-inoculated 5-week-old Col-0 and *mlo2 mlo6 mlo12* mutant plants with either water (mock control) or *A. laibachii* (isolate Nc14) zoospores and at 8-9 dpi challenged the pre-inoculated leaves with *G. orontii*. We then scored the penetration success of the powdery mildew pathogen at 48 hpi by microscopic analysis. We found (based on two independent experiments) that the pre-inoculation of plants with *A. laibachii* did not change the *G. orontii* entry levels. Col-0 plants remained fully susceptible (>92% host cell entry) under these conditions, while the triple mutant plants remained either fully (*mlo2*-5 *mlo6*-2 *mlo12*-1; 0% host cell entry) or nearly fully (*mlo2*-6 *mlo6*-4 *mlo12*-8; 2-6% host cell entry) resistant (Table 1). These values are equivalent to the figures obtained in standard *G. orontii* inoculation experiments with these genotypes (Fig. 1E). Thus, pre-inoculation with *A. laibachii* fails to break resistance conditioned by *mlo* triple mutants in Arabidopsis.

**Table 1:** *G. orontii* host cell entry rates of Arabidopsis plants after mock treatment or pre-inoculation with *A. laibachii* isolate Nc14^1^.

Experiment	Genotype	Mock control (pre-treated with water)	Pre-inoculated with *A. laibachii* isolate Nc14
1	Col-0	98.9% ± 1.0%	96.4% ± 4.3%
	*mlo2*-5 *mlo6*-2 *mlo12*-1	0.0% ± 0.0%	0.0% ± 0.0%
	*mlo2*-6 *mlo6*-4 *mlo12*-8	6.1% ± 4.8%	4.1% ± 3.5%
2	Col-0	92.8% ± 1.8%	97.2% ± 1.2%
	*mlo2*-5 *mlo6*-2 *mlo12*-1	0.0% ± 0.0%	0.0% ± 0.0%
	*mlo2*-6 *mlo6*-4 *mlo12*-8	3.9% ± 2.4%	1.8% ± 1.4%

^1^Mean of the entry rate ± SD at 48 hpi based on data from six plants per genotype and condition. For each plant, typically ca. 200 interaction sites from at least three rosette leaves were scored.

## Discussion

We studied the infection phenotype of two *mlo2 mlo6 mlo12* triple T-DNA mutants based on independent allele combinations (*mlo2*-5 *mlo6*-2 *mlo12*-1 and *mlo2*-6 *mlo6*-4 *mlo12*-8) with a number of plant-colonizing microbes in a quantitative manner. These species, with the exception of the endophyte *S. indica*, are mostly pathogenic, comprised representatives from three kingdoms of life (bacteria, oomycetes, fungi) that utilize different trophic lifestyles (obligate biotrophs and hemibiotrophs) and diverse infection strategies. They thus constitute a broad panel of microbial species that can colonize the model plant *A. thaliana*.

As expected and previously shown for one of the two mutant lines^11^, both *mlo2 mlo6 mlo12* triple mutants were highly resistant against the adapted powdery mildew pathogen *G. orontii* (Fig. 1). We noted, however, that the newly generated *mlo2*-6 *mlo6*-4 *mlo12*-8 mutant showed a very low level of host cell entry (1–6%), whereas the previously reported *mlo2*-5 *mlo6*-2 *mlo12*-1 mutant was fully immune (0% host cell entry; Fig. 1E and Table 2). This minute yet reproducible difference in host cell penetration could be due to residual levels of truncated *MLO* mRNA in the *mlo2*-6 *mlo6*-4 *mlo12*-8 mutant. This concerns particularly *MLO6*, for which the T-DNA insertion site in the *mlo6*-4 mutant is at the very distal end of the gene (Fig. 1A). Partial functionality of C-terminally truncated protein variants was previously demonstrated in the case of barley Mlo^43^. Alternatively, anonymous second-site T-DNA insertions in the *mlo2*-6 *mlo6*-4 *mlo12*-8 mutant may modulate the powdery mildew infection phenotype in this line. Potent powdery mildew resistance of the *mlo2*-5 *mlo6*-2 *mlo12*-1 mutant line was previously also found with respect to another adapted powdery mildew fungus, the tomato pathogen *O. neolycopersici* ^15^. In addition, the triple mutant is also resistant to two non-adapted powdery mildew fungi, the pea pathogen *Erysiphe pisi* and *Blumeria graminis* f.sp. *hordei* (*Bgh*), a pathogen of barley^11^ (Table 2).

**Table 2:** Compilation of infection phenotypes of the Arabidopsis *mlo2*-5 single mutant and *mlo2 mlo6 mlo12* triple mutants^1^.

Microbial species (disease)	Strain/isolate	Type of microorganism	Mode of plant entry	*mlo2*-5	*mlo2*-5 *mlo6*-2 *mlo12*-1	*mlo2*-6 *mlo6*-4 *mlo12*-8	Reference
*Albugo laibachii* (white rust)	Norwich 14 (Nc14)	Oomycete (obligate biotroph)	Stomata	n.t.^2^	~	~	This study
*Alternaria alternata* (black spot)	2177/00	Fungus (necrotroph)	Stomata and/or direct penetration^4^	~	↑↑	n.t.	11
*Alternaria brassicicola* (leaf spot)	MUCL 20297	Fungus (necrotroph)	Stomata and/or direct penetration^4^	↑	↑↑	n.t.	11
*Blumeria graminis* f.sp. *hordei* (powdery mildew; non-adapted)	K1	Fungus (obligate biotroph)	Direct penetration^4^	↓	↓↓	n.t.	11
*Botrytis cinerea* (grey mould)	Camalexin-sensitive strain	Fungus (necrotroph)	Direct penetration^4^	↓↓	↓↓	n.t.	20
*Colletotrichum higginsianum* (anthracnose)	IMI349063	Fungus (hemibiotroph)	Direct penetration^4^	n.t.	↓	↓	This study
*Erysiphe pisi* (powdery mildew; non-adapted)	MPIPZ^3^ isolate	Fungus (obligate biotroph)	Direct penetration^4^	↓	↓↓	n.t.	11
*Fusarium oxysporum* (vascular wilt and root rot)	Fo5176	Fungus (hemibiotroph/necrotroph)	Wounding/cell junctions^5^	n.t.	↑	↑	This study
*Golovinomyces orontii* (powdery mildew, adapted)	MPIPZ isolate	Fungus (obligate biotroph)	Direct penetration^4^	↓	↓↓	↓↓	11 This study
*Hyaloperonospora arabidopsidis* (downy mildew)	Noco2	Oomycete (obligate biotroph)	Cell junctions^5^	n.t.	~	~	This study
*Oidium neolycopersici* (powdery mildew; adapted)	Wageningen	Fungus (obligate biotroph)	Direct penetration^4^	↓	↓↓	n.t.	15
*Phytophthora infestans* (late blight)	208 m2	Oomycete (hemibiotroph)	Stomata and/or direct penetration^4^	↑	↑↑	n.t.	11
*Serendipita indica* (syn: *Piriformospora indica*)	DSM11827	Fungus (endophyte)	Direct penetration^4^/cell junctions^5^	n.t.	~	~	This study
*Pseudomonas syringae* pv. *maculicola* (bacterial speck)	ES4326	Bacterium (hemibiotroph)	Stomata	n.t.	↑	↑	This study

^1^↑ somewhat enhanced disease symptoms/pathogen proliferation, ↑↑ strongly enhanced disease symptoms/pathogen proliferation, ~ roughly unaltered disease symptoms/pathogen proliferation, ↓ somewhat reduced disease symptoms/pathogen proliferation, ↓↓ strongly reduced disease symptoms/pathogen proliferation. ^2^n.t., not tested. ^3^MPIPZ, Max Planck Institute for Plant Breeding Research, Cologne. ^4^Direct penetration into epidermal cells. ^5^Not penetrating epidermal cells but entering through anticlinal cell-cell junctions.

In comparison to Col-0, both *mlo2 mlo6 mlo12* triple mutant lines exhibited unaltered infection phenotypes upon challenge with the obligate biotrophic oomycetes *H. arabidopsidis* and *A. laibachii* and following inoculation with the fungal endophyte *S. indica* (Figs 2, 3 and 7). Thus, mutations in genes *MLO2*, *MLO6* and *MLO12* apparently do not affect colonization of Arabidopsis by these microbes. However, we only tested one particular Arabidopsis accession (Col-0) with single strains/isolates of the respective microorganisms and performed the assays under standard laboratory conditions. In addition, only certain parameters of plant colonization such as successful sporulation (*H. arabidopsidis* and *A. laibachii*) or the amount of fungal biomass (*S. indica*) were assessed. Therefore, we cannot rule out that the respective infection phenotypes of *mlo2 mlo6 mlo12* mutant plants might be altered in different settings or upon deployment of other host or microbial genotypes.

In contrast to the examples outlined above, infection phenotypes with the fungal pathogens *C. higginsianum* and *F. oxysporum* and the bacterial pathogen *P. syringae* were overall consistently affected in both *mlo2 mlo6 mlo12* mutant lines. In the case of *F. oxysporum* and *P. syringae*, the triple mutant plants showed enhanced disease symptoms as exemplified by either extended tissue chlorosis and necrosis (*F. oxysporum*; Fig. 5) or more pronounced leaf chlorosis (*P. syringae*; Fig. 6). In the case of *F. oxysporum*, a pathogen that colonizes plants *via* roots^44^, the enhanced disease symptoms could be caused by both root or shoot effects of the *mlo* triple mutant. Since at the onset of symptom development (5–7 dpi; Fig. 5A) the fungus typically has reached shoot tissue^45^, we are at present unable to discriminate between these two possibilities. For *P. syringae* we recorded about two-fold higher bacterial titres in the mutants (Fig. 6). This contrasts with the situation for *C. higginsianum*, where we found lower levels of host cell penetration with the fungal pathogen in mutant plants (Fig. 4). Thus, infection phenotypes of virulent pathogens can be modulated in either direction, towards enhanced susceptibility (as observed for *F. oxysporum* and *P. syringae*) or towards increased resistance (as found for *G. orontii* and *C. higginsianum*) in *mlo2 mlo6 mlo12* mutant plants (Table 2). The higher titres of *P. syringae* pv. *maculicola* strain ES4326 in the triple mutant were somewhat unexpected since a previous study reported enhanced resistance of *mlo2*-6 and *mlo2*-7 single mutant plants upon challenge with *P. syringae* pv. tomato DC3000^46^. The seeming discrepancy might be explained by the usage of different bacterial pathovars and/or differences between *mlo2* single and *mlo2 mlo6 mlo12* triple mutant plants. Notably, except maybe for *C. higginsianum* (Fig. S2), the two distinct *mlo2 mlo6 mlo12* mutant lines behaved in an indistinguishable manner in response to the various pathogenic and endophytic microbes, suggesting that the observed phenotypes reflect the authentic effect of the *mlo2 mlo6 mlo12* mutations and are not caused by any second-site mutations in these lines.

The data of the present study complement former analyses of the *mlo2*-5 *mlo6*-2 *mlo12*-1 mutant line with an extended range of pathogens. Previously, we reported enhanced powdery mildew resistance (*G. orontii*, *O. neolycopersici*, *E. pisi*, *Bgh*), but also enhanced disease symptoms and/or an increased pathogen biomass upon challenge with the adapted necrotrophic fungi *A*. *alternata* and *A. brassicicola* and the non-adapted hemibiotrophic oomycete *P*. *infestans* ^11^ (Table 2). Likewise, we discovered apart from powdery mildews one further example of enhanced disease resistance for the *mlo2*-5 *mlo6*-2 *mlo12*-1 mutant. This was a camalexin-sensitive isolate of the necrotrophic fungal pathogen *Botrytis cinerea*, which caused less disease symptoms on the mutant line than on Col-0 wild-type plants^20^ (Table 2). In most of these cases, the *mlo2*-5 *mlo6*-2 *mlo12*-1 triple mutant showed stronger alterations of the infection phenotype than the respective *mlo2*-5 single mutant, suggesting a general cooperative effect of the three Arabidopsis *MLO* genes in modulation of immunity (Table 2). In the present work, we extended these former studies conducted with only one *mlo2 mlo6 mlo12* triple mutant, which are less conclusive due to the presence of potential second-site mutations in the genetic background of a single line. In addition, most of the previous pathogen assays lacked quantification; thus the present dataset is more complete and robust than the former analyses.

Apart from the Arabidopsis *mlo2 mlo6 mlo12* triple mutants studied here in detail, the barley *mlo* mutant has been most extensively investigated regarding the infection phenotypes with phytopathogens other than powdery mildew (*Bgh*). It was for example reported that interactions with obligate biotrophic rust fungi (leaf rust, *Puccinia hordei*; stripe rust, *Puccinia striiformis*; stem rust, *Puccinia graminis* f.sp. *tritici*), the take-all fungus (*Gaeumannomyces graminis*) and the scald fungus (*Rhynchosporium secalis*) were not affected by mutations in barley *Mlo* ^47^. In that study it was, however, noted that the premature leaf senescence phenotype of *mlo* mutants, which is associated with necrotic leaf spotting, may somewhat reduce the extent of colonization by rusts (i.e., necrotic areas not being colonized by these obligate biotrophic fungi). Though that report provides valuable information regarding a number of common barley diseases, it rests in part on “personal communications” and lacks any cytological assessment and/or quantitative data.

In contrast to these pathogens, leading to seemingly unaltered infection phenotypes, a number of phytopathogens have been described that show an aberrant outcome of colonization on barley *mlo* genotypes. A prominent example is the interaction of barley with the rice blast fungus, *Magnaporthe oryzae*. While *Mlo* wild-type plants show limited colonization by the fungal pathogen, *mlo* mutant plants are hyper-susceptible to *M. oryzae* ^48^. In addition, *mlo* mutant lines display more severe disease symptoms than *Mlo* genotypes in response to *Ramularia collo-cygni*, the causal agent of *Ramularia* leaf spot disease^49^. Furthermore, it has been reported that the mutant shows accelerated spread of the fungal pathogen *Fusarium graminearum* in infected ears^50^. However, a recent study did not find an increased DNA content of the latter two pathogens on barley *mlo* genotypes upon natural infections in the field^51^. An interesting example of a seemingly developmentally controlled effect of *mlo* mutations on infection by the hemibiotrophic pathogen *Phytophthora palmivora* has been reported lately^52^. The authors of this study found that the oomycete shows delayed colonization of young tissues in barley *mlo* plants. In addition to encounters with pathogens, the interaction with the beneficial mycorrhizal fungus *Funneliformis mosseae* (syn. *Glomus mosseae*) also seems to be affected in barley *mlo* mutant plants. Colonization intensity and arbuscule abundance were found to be reduced in a *mlo* null mutant compared to its isogenic parental *Mlo* wild type line^53^.

Based on the recent study, reporting that pre-infection of Arabidopsis with *A. laibachii* can break non-host resistance against the Irish famine pathogen, *P. infestans* ^42^, we explored whether pre-inoculation of our *mlo* triple mutant lines with *A. laibachii* can likewise result in a breakdown of *mlo* resistance. We found that pre-inoculation with *A. laibachii* did not affect the resistance phenotype of *mlo2 mlo6 mlo12* triple mutant plants under our conditions (Table 1). This outcome is consistent with the fact that *A. laibachii* also failed to overcome non-host resistance of Arabidopsis against the barley powdery mildew pathogen^42^. Maybe the defence suppression exerted by the white blister rust pathogen is not effective in the leaf epidermis, the target tissue of powdery mildew pathogens. Alternatively, or in addition, the lack of compatibility conferred by *mlo* triple mutants might be too powerful to be disabled by *A. laibachii*. This would be in line with the results of recent genetic analysis, demonstrating that single or even multiple defence pathways are dispensable for resistance of the *mlo2*-5 *mlo6*-2 *mlo12*-1 triple mutant^54^.

Taken together, mutations in genes *MLO2*, *MLO6* and *MLO12* in Arabidopsis and *Mlo* in barley, which condition broad-spectrum powdery mildew resistance in these species, also modify the outcome of infections with some other, but not all, microbial species (Table 2). This situation is consistent with an authentic and broadly acting role of the respective MLO proteins in plant immunity. Data from co-expression networks support such a role for MLO proteins in immunity^55^. The changed infection phenotypes could thus be the result of perturbed regulation of immunity in the absence of MLO proteins, which may be to the benefit of some microbes and to the disadvantage of others.

It remains, however, puzzling that no consistent pattern is recognizable on first view regarding the altered infection phenotypes. The tested microbes belong to different kingdoms of life (bacteria, oomycetes and fungi) and engage in different modes of plant colonization and trophic lifestyles (obligate biotrophs, hemibiotrophs and necrotrophs). We found a tendency for enhanced susceptibility in *mlo2 mlo6 mlo12* mutant plants to correlate with a hemibiotrophic/necrotrophic lifestyle (Table 2). The exception from this trend is the hemibiotrophic fungus *C. higginsianum*, which shows reduced host cell entry on *mlo2 mlo6 mlo12* genotypes (Fig. 4B). An obvious commonality in the mode of infection between powdery mildews and *C. higginsianum* is that both phytopathogens directly enter epidermal host cells *via* appressoria/infection pegs that breach the cuticle and underlying cell wall (refs 27, 56 and Table 2). By contrast, leaf-colonizing *A. laibachii* and *P. syringae* enter host tissue through stomata^26, 57^, while *H. arabidopsidis* penetrates *via* anticlinal cell walls of neighboring epidermal pavement cells^24^. The remaining two microbes, *F. oxysporum* and *S. indica*, gain access to plant tissues *via* the roots^34, 44^, which lack a cuticle. Thus, the mode of host cell entry might be a key factor determining the modulation of the infection phenotype on *mlo2 mlo6 mlo12* mutant plants. This notion is largely consistent with results from previous patho-assays with this mutant (Table 2). *A. alternata*, *A. brassicicola* and *P. infestans* may be considered as possible exceptions in this respect. However, these pathogens have been reported to invade by both entry through stomata and direct host cell penetration, depending on environmental factors such as temperature and humidity^58, 59^.

It nevertheless remains unclear why *mlo* mutants typically show a spectacular level of resistance against powdery mildews, while infection phenotypes to other pathogens are generally only mildly impacted. One possibility is that powdery mildew fungi actively target MLO proteins for defence suppression, as previously suggested^60, 61^, or interfere with membrane dynamics or vesicle trafficking for compatibility^62^. Notably, it also appears that *mlo* genotypes of some plant species (such as Arabidopsis and barley) show altered interactions with pathogens apart from powdery mildews, whereas in other plant species (e.g. pea and tomato) this seems not to be the case^14, 15^. It thus remains a future task to disentangle which molecular parameters determine whether a microbial species shows a modified infection phenotype on *mlo* plants and why some plant species are affected by this phenomenon and others are not.

## Materials and Methods

### Plant materials

Triple mutants *mlo2*-5 *mlo6*-2 *mlo12*-1 and *mlo2*-6 *mlo6*-4 *mlo12*-8 were generated by crossing and are based on the following T-DNA insertion single mutants (all in the genetic background of accession Col-0): *mlo2*-5 (SAIL_878_H12), *mlo2*-6 (SALK_050191), *mlo6*-2 (SAIL_0523_D09), *mlo6*-4 (SALK_039680), *mlo12*-1 (SLAT 24-21) and *mlo12*-8 (SALK_041042). The *mlo2*-5 *mlo6*-2 *mlo12*-1 mutant has been described before^11^. In addition to the *mlo* triple mutant lines, Arabidopsis accessions Columbia (Col-0), Landsberg *erecta* (L*er*) and Keswick (Ksk-1) as well as mutants *edr1* ^41^, *eds1*-2^36^, *npr1*-1^63^, *pmr4-*1^10^, *sid2-*1^64^ and *sid2-*2/*eds16*-1^40^ were used in this study. All mutant lines are in the *Arabidopsis thaliana* Col-0 background.

### RT-PCR analysis

Total RNA was extracted from rosette leaves of 6-week-old plants using the Nucleospin RNA plus kit (Macherey-Nagel, Düren, Germany). cDNA was generated from total RNA (1 µg) with the High Capacity RNA-to-cDNA kit (Thermo Fisher Scientific, Waltham, MA, USA) following the manufacturer’s instructions. RT-PCR analysis (35 cycles) was performed with the following primer pairs: *AtMLO2* (2 C 5′-ATG GAG ATG GAG ATA AAC CCG GTC-3′ and 2bw1 5′-ACT AGT ATC TAG GAG AAG GAG-3′; amplicon size ca. 1.2 kb), *AtMLO6* (6 G 5′-GCT TTC TTT GTC TGG AGT ACG-3′ and 6B4 5′-CAA GAA CTG GTT TCA TTT AGC-3′; amplicon size ca. 0.65 kb) and *AtMLO12* (18 K 5′-AAA GTA GCA TTA GTA TCT GCC-3′ and 18 L 5′-ATG TCT TCT GTT TTG TGG TGG-3′; amplicon size ca. 0.8 kb).

### *G. orontii* infection assays

Plants were grown under short-day conditions (8 h light, 22 °C; 16 h dark, 20 °C; 80 μmol m^−2^ s^−1^) and inoculated at the age of six weeks. Heavily *G. orontii*-infected leaves of accession Col-0 were used for leaf-to-leaf contact inoculation. Samples for microscopy were taken at 48 hpi and cleared in destaining solution (1:2 mixture of stock solution and ethanol; stock solution 1:2:1 mixture of lactic acid, glycerol and deionized H_2_O) at room temperature. Fungal structures were visualized with Coomassie Brilliant Blue staining (0.6% Coomassie Brilliant Blue R-250 (Carl Roth GmbH, Karlsruhe, Germany) in ethanol) by briefly dipping the specimens into the staining solution shortly prior to mounting the samples for microscopy. For quantification of fungal host cell entry, the proportion of germinated fungal sporelings that developed secondary hyphae served as an approximation of penetration success.

### *H. arabidopsidis* infection assays

*H. arabidopsidis* isolate Noco2 was kindly provided by Jane Parker (Max Planck Institute for Plant Breeding Research, Cologne, Germany) and maintained on Arabidopsis Col-0 by weekly transfer to healthy 14- and 21-day-old seedlings. For inoculation, healthy 16-day-old seedlings were evenly sprayed with a spore suspension (4 × 10^4^ spores ml^−1^). Plants were allowed to dry and sprayed once more with the spore suspension. After drying, plants were kept under a sealed lid (100% relative humidity) in a growth chamber at 16 °C with a 10 h photoperiod (100 μmol m^−2^ s^−1^). Seven days after inoculation, aerial parts of the plants were harvested and the fresh weight (FW) was determined. Evaluation of *H. arabidopsidis* propagation was performed by quantifying conidiospores with a hemocytometer as described^65^ and represented as the number of conidiospores per gram FW. For normalization, the corresponding value for Col-0 was set as 100%. L*er* and *eds1*-2 plants were used as resistant and super-susceptible controls, respectively.

### *A. laibachii* infection assays

Plants were grown in short-day conditions (10 h light, 22 °C, 65% humidity/14 h dark, 21 °C, 60% humidity, 40 μmol m^−2^ s^−1^) and inoculated at the age of six weeks. *A. laibachii* (isolate Nc14^66^) zoospores obtained from propagation on Arabidopsis accession Ws-0 were suspended in water (10^5^ spores ml^−1^) and incubated on ice for 30 min. The spore suspension was filtered through Miracloth (Calbiochem, San Diego, CA, USA) and sprayed onto the plants using a spray gun (~700 μl/plant), both on the adaxial and abaxial sides of rosette leaves. Plants were incubated at 8 °C in a cold room in the dark overnight. Inoculated plants were kept under 10 h light/14 h dark cycles with a 20 °C day and 16 °C night temperature. Infection rates were determined at 10 dpi for 5–6 individuals per genotype by counting numbers of infected *versus* uninfected leaves.

### *C. higginsianum* infection assays

For *C. higginsianum* infection assays, cultures of the fungal isolate IMI349063 and Arabidopsis plants were grown as described previously^67^. Plants were spray-inoculated with conidial suspension (5 × 10^5^ spores ml^−1^), covered in sealed propagators to maintain 100% humidity and incubated in a controlled environment (16 h photoperiod, 80 μmol m^−2^ s^−1^, 25 °C). Methods for Trypan blue-lactophenol staining of infected leaves and subsequent clearing of the stained tissues in chloral hydrate were adapted from^68^. To quantify the frequency of appressorial penetration, Arabidopsis leaves were cleared in ethanol:chloroform (3:1) and mounted on slides in lactophenol. Appressorium-based penetration was scored as the presence of a visible hypha in the underlying epidermal cell at 3 dpi.

### *F. oxysporum* infection assays

Arabidopsis plants used for inoculation with *F. oxysporum* were grown in controlled conditions with 11 h light/13 h darkness (90 μmol m^−2^ s^−1^) at 22 °C. Two-week-old seedlings were uprooted from soil, cleaned with tap water and placed in a spore suspension of *F. oxysporum* (isolate Fo5176^69^) for 5 min. Spores were harvested from a 5-day-old liquid culture^38^ by filtering through three layers of Miracloth. Spores were resuspended in tap water to 10^6^ spores/ml for Arabidopsis inoculation. Autoclaved tap water was used for mock treatment. Inoculated and mock-treated seedlings were replanted into the original soil, placed under a transparent plastic hood to maintain humidity, and grown under controlled conditions with 11 h light/13 h darkness (28 °C, 70% relative humidity). The disease index was scored at 5, 7 and 10 dpi on a scale ranging from 0 to 5 (0 = no disease symptoms; 1 = 1–2 leaves with yellow veins or plant is smaller; 2 = some fully developed leaves show chlorosis or yellow veins; 3 = most fully developed leaves show chlorosis; 4 = all fully developed leaves show chlorosis; 5 = dead; adapted from^38^).

### *S. indica* infection assays

*Serendipita indica* (syn. *Piriformospora indica*) isolate DSM11827^33^ was obtained from the German collection of microorganisms and cell cultures in Braunschweig (Germany). The fungus was grown on complete medium^70^ supplemented with 1.5% agar at 24 °C in the dark. Arabidopsis plants were grown under sterile conditions (ca. 60 seedlings per plate) in a growth cabinet (120 μmol m^−2^ s^−1^; 10 h light; 22 °C/18 °C (light/dark); 60% humidity) on vertically placed square petri dishes containing growth medium (5 mM KNO_3_, 2.5 mM KH_2_PO_4_, 3 mM MgSO_4_, 3 mM, Ca(NO_3_)_2_, 50 μM Fe-EDTA 70 μM H_3_BO_3_, 14 μM MnCI_2_, 0.5 μM CuSO_4_, 1 μM ZnSO_4_, 0.2 μM NaMoO_4_, 10 μM NaCl, 0.01 μM CoCl_2_), modified as described^71^ and supplemented with 0.45% Gelrite (Duchefa Biochemie, Haarlem, The Netherlands). Roots of 2-week-old plants were inoculated with 5 × 10^5^ *S. indica* chlamydospores (1 ml per petri dish). The inoculated roots were harvested at 3 and 7 days after inoculation for subsequent DNA extraction. Genomic plant and fungal DNA was obtained as described^72^. qRT-PCR analysis was performed on a Bio-Rad iCycler iQ5 using a standard protocol. Forty ng of genomic DNA served as template with 20 µL of SYBR Green JumpStart Taq ReadyMix (Sigma-Aldrich, Taufkirchen, Germany) and 350 nM oligonucleotides. Fungal colonization was determined by the comparative *C*_T_ method^73^ by subtracting the raw cycle threshold values of *S. indica Internal Transcribed Spacer* (5′-CAACACATGTGCACGTCGAT-3′/5′-CCAATGTGCATTCAGAACGA-3′) from those of *AtUbi5* (At3g62250; 5′-CCAAGCCGAAGAAGATCAAG-3′/5′-ATGACTCGCCATGAAAGTCC-3′).

### *P. syringae* infection assays

Arabidopsis seeds were stratified and then grown on soil (Dachstaudensubstrat SoMi 513; Hawita). Growth conditions for experiments 1-4 were: 10 h day (80 μmol m^−2^ s^−1^)/14 h night cycle, 21 °C, and a relative humidity of 68%; experiment 5: 10 h day (80 μmol m^−2^ s^−1^) at 21 °C/14 h night cycle at 18 °C, and a relative humidity of 68%. For experiment 5, the soil was sterilized by microwaving for 10 min before usage. Fourteen-day-old healthy seedlings were individually transferred to bigger pots (9 × 9 × 8 cm).

*P. syringae* pv. *maculicola* strain ES4326 carrying the *luxCDABE* operon from *Photorhabdus luminescens* under the control of a constitutive promoter (*Psm lux* ^39^; kindly provided by Prof. Jürgen Zeier, Heinrich Heine University Düsseldorf, Germany) was cultivated at 28 °C in King’s B medium supplemented with 50 μg ml^−1^ rifampicin and 25 μg ml^−1^ kanamycin. Overnight log phase liquid cultures were diluted to an OD_600_ of 0.001 (experiments 1 and 2) or 0.0005 (experiments 3-5) in 10 mM MgCl_2_. Leaf inoculations were performed with 5-week-old plants of uniform, healthy appearance. Using a needleless 1 ml syringe, three mature leaves per plant were infiltrated from the abaxial side. The bacterial growth in the leaves was determined *via* the bioluminescence of the *Psm lux* stain at 0 dpi (following infiltration) and at 3 dpi. The measurement was carried out on leaf discs (r = 3 mm, adaxial side up) from inoculated and control leaves, individually placed in 250 µl MgCl_2_ in 96-well microtiter plates (flat bottom). The bioluminescence of each leaf disc was monitored as a 10 s averaged measurement per well with a 10 s delay at the start of each plate using the ‘Centro XS³ LB 960 Microplate Luminometer’ and the corresponding ‘MikroWin 2000 software’ (Berthold Technologies, https://www.berthold.com/). The average auto-luminescence (background of untreated leaves) was subtracted from the luminescence values of the treated leaves, and the average of three leaf discs per plant was determined. Increasing luminescence values (indicated by relative light units, RLU) correspond to increasing bacterial titres and reflect bacterial growth determined by the standard plate assay^39^.

### Statistical analysis

Statistical analyses were carried out using R v3.3.2 software for Windows^74^ (http://www.r-project.org/). Owing to limited sample size, non-normal distribution and unequal variance, we performed non-parametric statistical tests. Datasets for *G. orontii*, *F. oxysporum*, *C. higginsianum*, *S. indica* and *P. syringae* were analysed by Generalized Linear Modeling (GLM), assuming Poisson or quasi-Poisson distribution (continuous datasets) or binomial distribution (ratio/percentage datasets), as recommended for this type of data^75^. The other datasets (*H. arabidopsidis* and *A. laibachii*) were additionally analysed by Wilcoxon-Mann-Whitney rank sum test.

### Data availability

All data generated or analysed during this study are included in this published article (and its Supplementary Information files). Respective raw data are available from the corresponding author upon request.

## Electronic supplementary material

Supplementary Information
