# Supplementary material for: The powdery mildew-resistant Arabidopsis mlo2 mlo6 mlo12 triple mutant displays altered infection phenotypes with diverse types of phytopathogens
Source: Sci Rep. 2017 Aug 24;7:9319. doi: 10.1038/s41598-017-07188-7 (PMC5570895; doi:10.1038/s41598-017-07188-7)

Title

The powdery mildew-resistant Arabidopsis *mlo2 mlo6 mlo12* triple mutant displays altered infection phenotypes with diverse types of phytopathogens

Authors

Johanna Acevedo-Garcia[1], Katrin Gruner[1], Anja Reinstädler[1], Ariane Kemen[2], Eric Kemen[2], Lingxue Cao[3], Frank L. W. Takken[3], Marco U. Reitz[4], Patrick Schäfer[4], Richard J. O´Connell[5], Stefan Kusch[1], Hannah Kuhn[1] and Ralph Panstruga[1]

Supplemental Figures

Figure S1. Data of a second infection experiment with *C. higginsianum*. Host cell entry rates of Col-0, *mlo2*-5 *mlo6*-2 *mlo12*-1 and *mlo2*-6 *mlo6*-4 *mlo12*-8 at 3 dpi with *C. higginsianum* (isolate IMI349063A). Plants were spray-inoculated with spore suspension (5×10^5 spores ml^{-1}). Data show the mean ± SD from counts of at least 140 appressoria from each leaf (one leaf each from of 3 different plants), i.e. at least 420 appressoria per plant genotype. Letters indicate statistically different groups (at least $P<0.05$) according to a GLM test (Poisson distribution).

Figure S2. Data of two additional infection experiments with *F. oxysporum*. Infection phenotypes were scored at 5, 7 and 10 dpi by assigning a disease index on a 0 (no symptoms) to 5 (severe disease symptoms) scale. Data shown are from a representative experiment and based on 15-20 seedlings per genotype. Each symbol in the categorical scatter plot (circle, square or triangle) represents the infection phenotype of one seedling. The crosses indicate the mean values ± SEM. * and ** indicate statistically significant differences from Col-0 ($P<0.05$ and $P<0.01$, respectively) according to a GLM test (Poisson distribution).

Figure S3. Data of four additional infection experiments with *P. syringae*. Five-week-old Arabidopsis plants were pressure-infiltrated with *P. syringae* pv. *maculicola lux* (OD_{600} = 0.001 in experiments 1 and 2 and OD_{600} = 0.0005 in experiments 3 and 4) and luminescence (RLU cm^{-2}; corresponding to bacterial titre) was determined at 3 dpi. The mutants *pmr4*-1, *sid2*-1 and *npr1*-1, included as additional controls in experiment 4, were previously reported to exhibit enhanced resistance [1] and susceptibility to *P. syringae* [2,3], respectively. **A** The boxplot shows data from four experiments (Exp. 1-Exp. 4) based on n = 7 to 13 plants per genotype, with each plant value represented by the median of three leaves. Centre lines mark the medians, upper and lower box limits indicate the 25th and 75th percentiles, respectively; upper and lower whiskers extend 1.5 times the interquartile range from the 25th and 75th percentiles, respectively; and dots represent outliners. Letters indicate statistically different groups (at least $P<0.05$) according to a GLM test (quasi-Poisson distribution). **B** Representative macroscopic infection phenotypes from experiment 4 at 3 dpi. Yellow arrows indicate the inoculated leaves.

References

1. Flors, V. *et al.* Interplay between JA, SA and ABA signalling during basal and induced resistance against *Pseudomonas syringae* and *Alternaria brassicicola*. *Plant J.* **54,** 81–92 (2008).
2. Cao, H. Bowling, S. A. Gordon, A. S. & Dong, X. N. Characterization of an Arabidopsis mutant that is nonresponsive to inducers of systemic acquired resistance. *Plant Cell* **6,** 1583–1592 (1994).
3. Nawrath, C. & Metraux, J. P. Salicylic acid induction-deficient mutants of Arabidopsis express *PR-2* and *PR-5* and accumulate high levels of camalexin after pathogen inoculation. *Plant Cell* **11,** 1393–1404 (1999).

Supplemental Figure 1

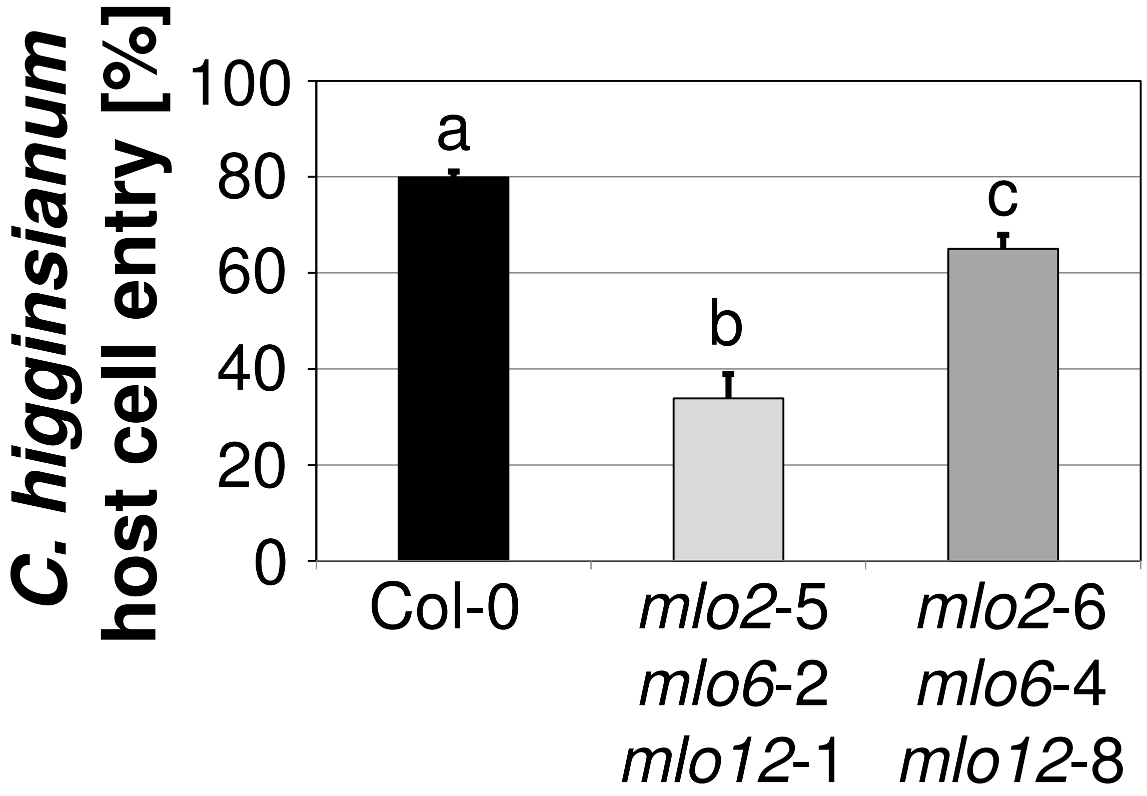

Supplemental Figure 2

A

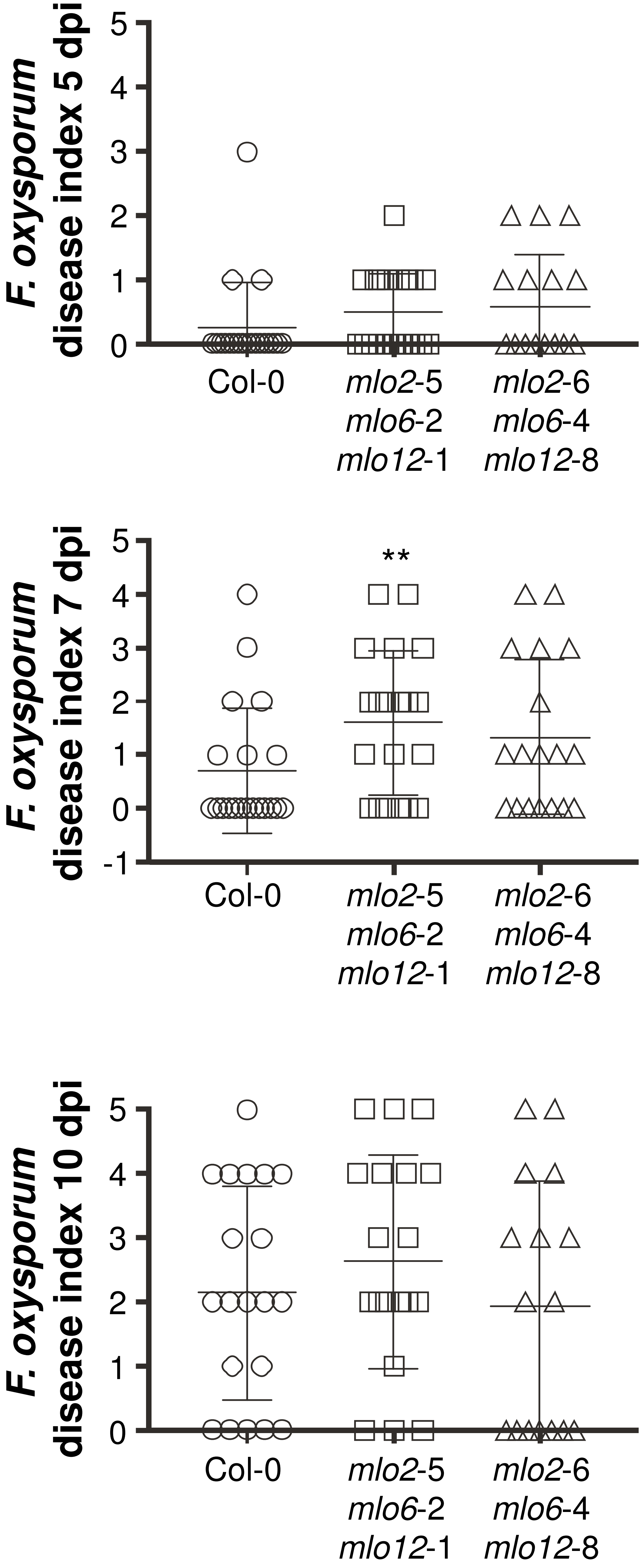

Supplemental Figure 2

B

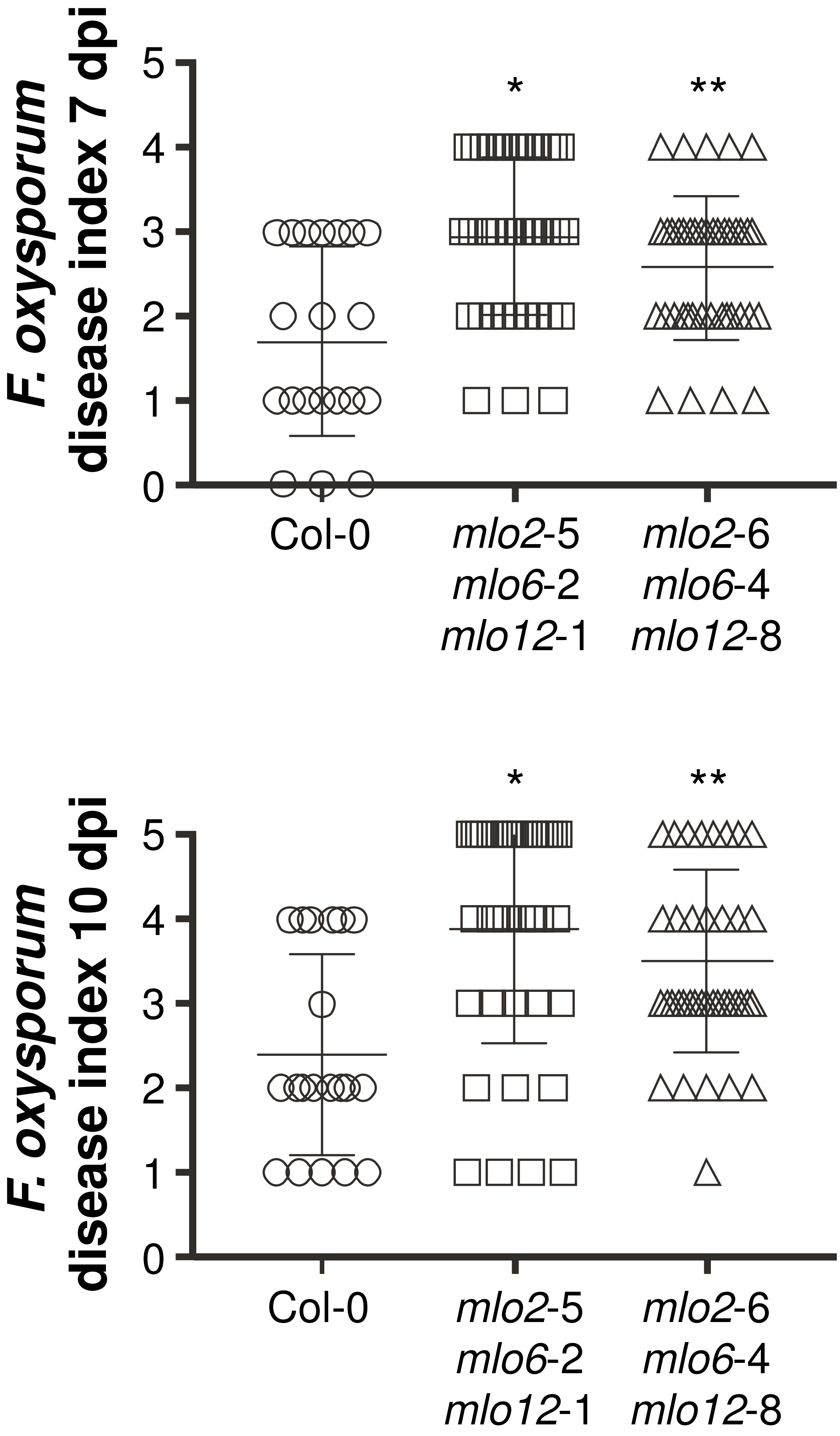

Supplemental Figure 3

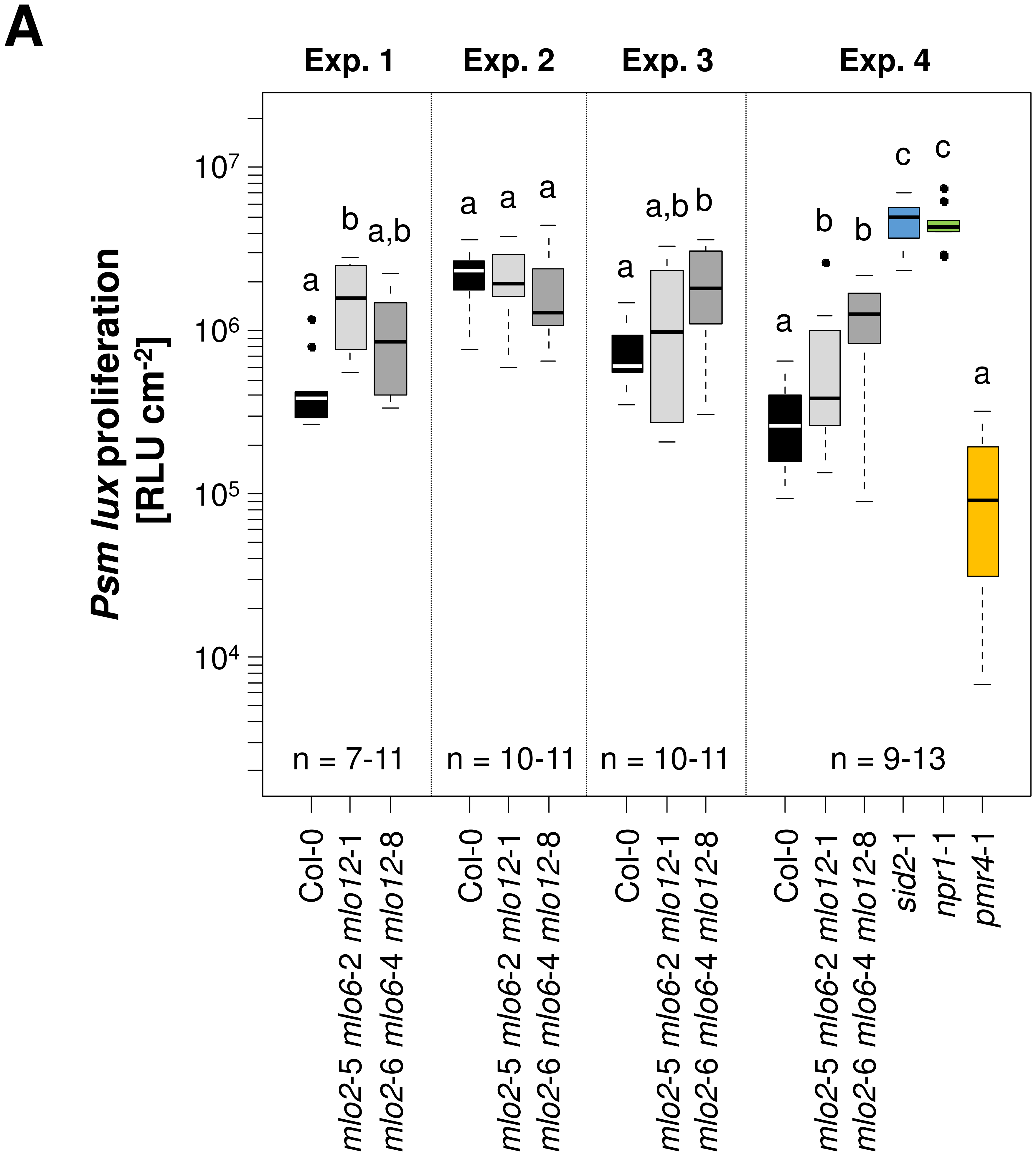

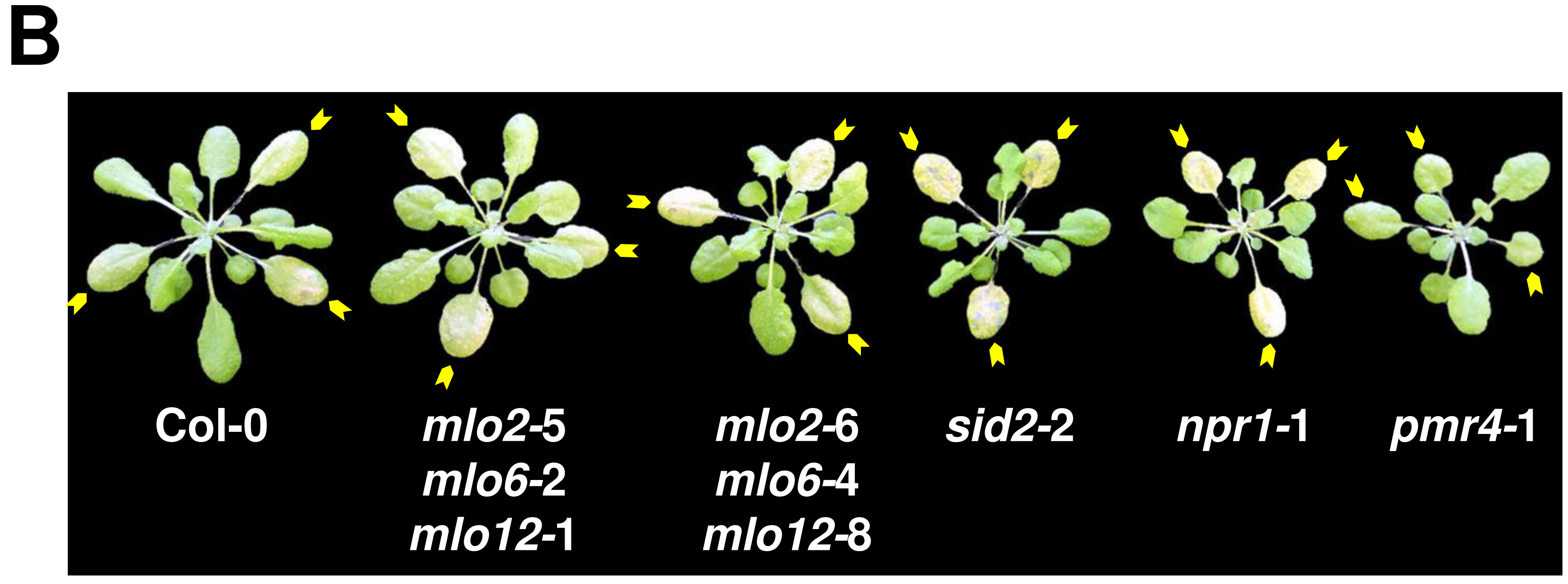

Supplement: Supplementary file 1 — Supplementary Information [file 41598_2017_7188_MOESM1_ESM.pdf]
